# Supplementary material for: Determining the Degree of [001] Preferred Growth of Ni(OH)2 Nanoplates
Source: Nanomaterials (Basel). 2018 Nov 30;8(12):991. doi: 10.3390/nano8120991 (PMC6316123; doi:10.3390/nano8120991)
Supplement: Supplementary file 1 [file nanomaterials-08-00991-s001.pdf]

Supplementary Materials

Determining the Degree of [001] Preferred Growth of $Ni(OH)_2$ Nanoplates

Taotao Li [1], Ning Dang [2], Wanggang Zhang [1], Wei Liang [1,*] and Fuqian Yang [3,*]

1. College of Materials Science and Engineering; Shanxi Key Laboratory of Advanced Magnesium-based Materials, Taiyuan University of Technology, Taiyuan, 030024, China
2. Laboratoire de Chimie Physique et Microbiologie pour les Matériaux et l'Environnement (LCPME), UMR 7564, CNRS-Université de Lorraine, Villers-lès-Nancy 54600, France
3. Materials Program, Department of Chemical and Materials Engineering, University of Kentucky, Lexington, KY 40506, USA

* Correspondence: liangwei@tyut.edu.cn (W.L.); fyang2@uky.edu (F.Y.)

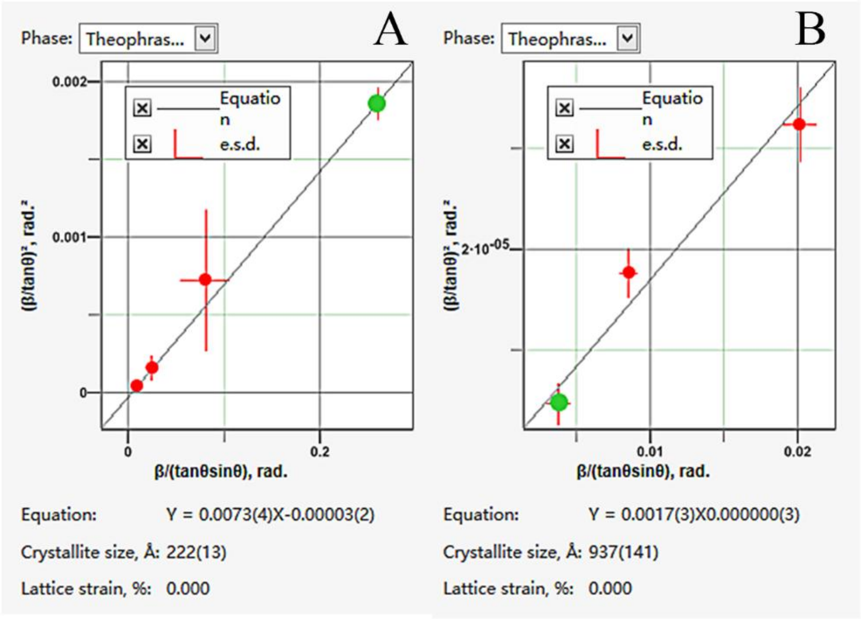

Figure S1. The simulated linear function with Halder-Wagner's method. The Diameters of D_{001} and D_{hk0} are determined.
